# Mental and Physical Work

**Published:** 1891-07

**Authors:** 


### MENTAL AND PHYSICAL WORK.

During the whole of my medical experience, says Professor Nussbaum, a celebrated Munich doctor, I have met with but few patients who had been made ill by overstraining their bones or their muscles; but, on the other hand, I have had to treat many hundreds of very serious cases of illness brought on by mental over-work—illnesses which were often most troublesome and difficult to cure. I am thoroughly convinced that the human body was not intended for the study-table, but for manual labor. I have always found that the healthiest and cheerfullest people are those who work in fields and gardens, and move about the greater part of the day in the fresh air. How different is the health of men who spend their time in offices. All of these usually suffer from cold feet, hot heads, and weak digestions—there are very few who do not constantly complain of nervous excitement. We all know that the exercise of any organ causes the blood to flow more copiously to it, and that its veins become thereby enlarged. This applies to the brain. When it acquires a greater flow of blood it does so at the expense of other organs. The arms, hands, and feet become poorer in blood as the brain becomes richer. The earlier such a state of things comes into action in the human body, the younger the individual is, the more damaging are the consequences of the balance thus faultily adjusted. A wretched future lies before the man whose mind is overworked while he is still a child. It is a thoroughly mistaken notion to believe that a child nine years old learns more in seven or eight hours a day than in four or five hours. Children should be in bed by nine o'clock, and should not be allowed to get up before five or six, otherwise the brain does not get enough rest. I hold that the principle of keeping a child occupied the whole day is an excellent one ; only a large proportion of the time should be devoted to bodily exercise, to the education of limbs and muscles, and, whenever it is possible, in the open air.

